# Bromides of Ammonium and of Potassium

**Published:** 1866-03

**Authors:** J. B. Read


### SELECTIONS.

### *Bromides of Ammonium and of Potassium.* By J. B. Read, M. D.

The use of Bromine and its compounds has of late years received much attention from physicians abroad. And two of its compounds are now, by daily increasing testimony, assigned high places in the list of remedies useful in the treatment of nervous diseases, and of many derangements of the uterus and its appendages. Bromine has heretofore been known to the profession in this country as the principal ingredient of "Bibrous antidote" for Rattlesnake poison, and during the war, is said to have been used by Surgeons in the Federal Hospitals as an application to gangrenous wounds. The Bromides of Ammonium and Potassium seem so nearly allied in their action on the economy, that one may be substituted for the other in the treatment of disease, and the results obtained by the use of one are equally applicable to the other. The action of these remedies is specially directed to the nervous system, on which most of their curative manifestations are shown.

Bromides produce a local anæsthetic action on the upper portion of the Trachial tube, and have a peculiar affinity to the uterus. They relieve pain, and have a decided hypnotic action, especially in cases of great excitement and sleeplessness, produced by long continued mental effort. In cases of this kind, Dr. Henry Behrend states that 25 grs. of the Bromide of Potassium, three times a day, will quiet the state of nervous excitement and produce sleep. These are cases in which our experience teaches us the various preparations of opium, and the drug itself increase rather than diminish the excitement. The Bromides are now freely used as sleep inducing agents in cases of Traumtaic excitement and delirium, and in the maniacal condition that sometimes attends on the puerperal state. 20 grs. doses of the Bromide of Potassium administered every two hours have recently, in my hands, produced decided effects in a case of this kind. When the patient came under my care she was suffering from a relapse of Puerperial mania of the most aggravated kind. Jagitalis was freely exhibited for some time with good effect, but had to be discontinued after a few days, and the case was then treated as above, and convalescence speedily set in. Up to this time, over 12 weeks, there has been no return of the trouble. The principal use, however, of these remedies up to this time, has been in the treatment of Hooping Cough and "Irritable uterus," with its chain of hemorrhages and unpleasant symptoms. To Dr. Gibbs, of Westminister Hospital, London, is due the credit of having first introduced the use of the Bromide of Ammonium as a remedy for Hooping Cough, and it has since been generally used and commented on. Dr. Ritchie (Braithwait's Retrospect, Jan. 1865) states that in Hooping Cough the general result has been to give relief to the spasmodic cough, the hoop, but that this result is not uniformly produced. Dr. Gibbs does not claim for it invariable success, but only that it is exceedingly valuable. During the epidemic of Hooping Cough which prevailed so extensively in this city during the past spring and summer, I have administered the remedy at all ages, and in all stages of the disease, and my experience teaches me that the observations of Dr. Halsey are correct.

1st. That the medicine is in itself harmless—that even in excessive doses it does not vomit or purge, but only induces a sleepy, drowsy state, and thus those that are not benefitted by its use are not injured by it.

2d. That it is especially useful in cases where the distressing hoop is incessant and violent. Many of these cases were relieved after a few doses of the medicine had been taken.

3d. That the Bromide was of no service where Bronchitis or Pneumonia existed, and these conditions had to be first removed by appropriate treatment before the Bromide was of any avail. In a case of Pertusiss, with convulsions, that was under my care during the summer, in a teething child of ten months, each hoop brought on a renewal of the convulsion, and this to such an extent that they had been uninterrupted for two hours before I saw the case. The Bromide of Ammonium was administered in 2 gr. doses every hour. After the second dose the spasms were quieted—the hooping occurred only at long intervals, and the child slept soundly—this sleep continued for six hours, and when the child awoke the hoop returned, but without a convulsion. The remedy was again given in 1 gr. doses, and its use continued for some days, gradually increasing the interval between the doses. The Pertussis was mastered, and the child recovered rapidly. It is especially in cases of this kind that it should be given when there is an urgent necessity for controlling the spasmodic cough, I should state, that, before giving the Bromide of Ammonium, I had resorted to full anæsthesia by chloroform, and that even when fully anasthetised, the hoop would come on. The medicine is not unpleasant to the taste, and may be given in combination with syrup of squills or Glycerine.

Dr. Gibbs gives 2 or 3 grs. three times daily to infants, and 4, 8, and 10 grs. as often to older children. Dr. Halsey mentions two striking cases of relief obtained by its use. He remarks that as a rule 1 gr. may be given as a dose for every year of the patient's age.

In the February number of Bro. Retros, page 231, Dr. G. Gorreyuer Griffith, of Dublin, treats at large of the use of the Bromide of Ammonium in Irritable uterus. He thinks it to be almost a true "Utero ovarain Specific." In cases of irritable uterus, at the monthly period, attended by great pain and excessive catamenial flow, he administered the Bromide of Ammonium as follows: 10 to 20 grs., if needed, every 4 hours, or more if required; and if it were desirable to act more promptly, and reduce the matritic discharge altogether and quickly, he uses the medicine thus, for the first dose, 20 to 50 or 60 grs., to be followed every hour or two by from 10 to 20 grs. This in cases where the hemorrhage is excessive.

Where pain is the prominent symptom, the drug may be administered in 20 gr. doses as often as may seem necessary. In cases where the disease assumes the paroxysmal type, Dr. Griffith prescribes 1 dr. to be taken at the very onset of the paroxysm. Where there is a periodic form ½ oz. or 1 scr. is given ten minutes before the time of attack, and continued afterwards, "pro re nata." The effect in such cases in my own experience with the drug, is rapid and satisfactory, more so than I have ever found from other remedial agents. Dr. Griffith writes: "The effect in allaying or removing pain, in checking, or altogether causing to cease, any uterine hemorrhage, is sometimes magical." He is unable to arrive at any definite conclusion as to the "manner in which the Bromide behaves itself in these cases. Acting both as an anodyne and a hamostotic agent at different times, the results of experience are positive."

In a case of excessive uterine hemorrhage, occurring in an old lady of sixty-four years, who had been previously treated with tr. Ferri. Mur. and other mineral hœmos tatics in vain, the use of the Bromide of Potassium speedily checked the bleeding, and at this time four months have elapsed without a recurrence of the trouble. The Bromide in the severe sick and neuralgic headaches that so constantly attend all uterine derangements, and even regular monthly periods are of great benefit, given in doses of 30 grs. three times a day. Dr. A. Garrod, F. R. S., states that he has used the Bromide of Potassium in many diseases, and has noticed the results of its action as follows:

1st. It produces no irritation of the mucus membrane of the nose or fauces.

2d. Some patients experience a peculiar sensation of dryness in the throat and neighboring parts.

3d. When given in large medicinal doses sleepiness or drowsiness and dull headache were occasionally noticed.

4th. When administered in very large amounts, some loss of power was noticed in the lower extremities, which passed off when the medicine was discontinued.

5th. The therapeutic action was decidedly what may be called alterative; that is, it relieved certain forms of syphilitic eruptions.

6. No action was observed upon the skin or kidneys. Sir Charles Locock states that he has found the Bromide of Potassium of service in severe cases of Hysterical Epilepsy, and other nervous affections connected with uterine diseases.

7th. Bromide of Potassium exerts a most powerful influence on the generative organs, lowering their function in a most remarkable degree.

8th. It is a remedy possessing many valuable powers, in diseases dependent on and accompanied by excitement of the generative organs, as Nymphomania, Priapism, and in the menorrhagia, that is apt to set in at the period of the menopaus.

9th. It produces an anæsthetic condition of the Larynx and

Pharynx, and hence has been usefully employed in examination of and operations on these parts.

Dr. R. McDonald directs attention to its use in certain forms of Epilepsy.

Dr. Ratcliffe says: "I can testify that this remedy was found more or less serviceable in cases the most dissimilar in character. So serviceable that the name of Sir Charles Locock ought to be remembered with gratitude by every Epileptic, and by many sufferers under other forms of convulsive diseases." Sir Charles Locock states that in fifteen cases of Hysterical Epilepsy he had only failed in one, and this one had fits not only at the time of menstruation, but in the intervals.

Dr. Brown Sequard also entertains the highest opinion of its efficiency. Mr. McDowell states that though at first much doubting the efficiency of the drug in these diseases, he has come to the conclusion that it has remarkable efficiency.

In the case of a lady who has been under my charge, and who has been subject to Epileptic attacks for the last six years, great benefit has resulted from the use of 20 gr. doses of the Bromide of Potassium three times daily. Under this treatment there has been no Epileptic seizure for four months. Besides these cases of Hysterical Epilepsy, reasoning from its known action on the generative organs, we should be led to expect good results from its use in Epileptic attacks, when they are traceable to sexual excess in males. While it is not claimed that these drugs are specifics for these affections, and that they will invariably relieve these diseases; still, the extraordinary results obtained by Sir Charles Locock, and the high opinion entertained of their power by the justly celebrated Brown Sequard and our own limited experience, lead us confidently to advise a full use and fair trial of the Bromides. Bromide of Potassium is administered in the same dose as the other Bromide.—*Savannah Journal of Medicine.*

